# Al_2_O_3_/ZrO_2_/Y_3_Al_5_O_12_ Composites: A High-Temperature Mechanical Characterization

**DOI:** 10.3390/ma8020611

**Published:** 2015-02-10

**Authors:** Paola Palmero, Giovanni Pulci, Francesco Marra, Teodoro Valente, Laura Montanaro

**Affiliations:** 1 Dipartimento di Scienza Applicata e Tecnologia, Politecnico di Torino, INSTM R.U. PoliTO, Laboratorio di Tecnologia ed Ingegnerizzazione dei Materiali Ceramici (LINCE), Corso Duca degli Abruzzi, 24, 10129 Torino, Italy; E-Mail: laura.montanaro@polito.it; 2 Dipartimento di Ingegneria Chimica Materiali e Ambiente, Sapienza Università di Roma, Laboratorio di Ingegneria dei Trattamenti Superficiali, INSTM, Via Eudossiana, 18, 00184 Roma, Italy; E-Mails: giovanni.pulci@uniroma1.it (G.P.); francesco.marra@uniroma1.it (F.M.); teodoro.valente@uniroma1.it (T.V.)

**Keywords:** Al_2_O_3_/ZrO_2_/YAG, elaboration, microstructure, high-temperature bending test, mechanical properties, thermal stability

## Abstract

An Al_2_O_3_/5 vol%·ZrO_2_/5 vol%·Y_3_Al_5_O_12_ (YAG) tri-phase composite was manufactured by surface modification of an alumina powder with inorganic precursors of the second phases. The bulk materials were produced by die-pressing and pressureless sintering at 1500 °C, obtaining fully dense, homogenous samples, with ultra-fine ZrO_2_ and YAG grains dispersed in a sub-micronic alumina matrix. The high temperature mechanical properties were investigated by four-point bending tests up to 1500 °C, and the grain size stability was assessed by observing the microstructural evolution of the samples heat treated up to 1700 °C. Dynamic indentation measures were performed on as-sintered and heat-treated Al_2_O_3_/ZrO_2_/YAG samples in order to evaluate the micro-hardness and elastic modulus as a function of re-heating temperature. The high temperature bending tests highlighted a transition from brittle to plastic behavior comprised between 1350 and 1400 °C and a considerable flexural strength reduction at temperatures higher than 1400 °C; moreover, the microstructural investigations carried out on the re-heated samples showed a very limited grain growth up to 1650 °C.

## 1. Introduction

As underlined by the recent scientific literature, there is an increasing interest towards new structural materials, able to maintain both stability and mechanical properties at high temperature.

Within the group of high-temperature ceramics, the nitrides and the oxides are by far the most important ones from a technological and industrial point of view [1]. In particular, ceramic oxides can be used in harsh environments, withstanding extremely severe operating conditions, such as high temperatures, oxidant and corrosive atmospheres [2]. This characteristic can enlarge their use in aeronautic and aerospace applications, such as for aircraft jet engines and high-efficiency power-generation gas turbines [3].

Recently, increasing attention has been paid to alumina-yttrium aluminum garnet (Y_3_Al_5_O_12_, YAG) composites, due to their good mechanical properties, high thermal stability and good chemical resistance [4]. Moreover, both single-crystal and polycrystalline YAG present excellent creep resistance, due to the large lattice parameter and, hence, large Burgers vector of the dislocations [5,6]. In 1995, Waku *et al.* first fabricated alumina/YAG eutectic composites for high-temperature structural applications [7], by melting the constituent raw oxides, followed by unidirectional solidification. This composite showed stable mechanical properties (*i.e.*, bending strength and creep resistance) and excellent chemical and microstructural stability from room temperature to 1820 °C [4,8]. Such good properties have been associated with the clean and strong interfaces between the constituents and the lack of grain boundaries and glassy phase [3].

Ever since, new eutectic compositions have been developed, such as the Al_2_O_3_/GdAlO_3_ [9] and the Al_2_O_3_/ZrO_2_ [2,10] systems. Since the beginning of the new century, the research on the eutectic materials shifted from Al_2_O_3_-based binary to ternary systems. In this frame, the ternary Al_2_O_3_/ZrO_2_/YAG eutectic composite is receiving much attention, due to its outstanding mechanical properties [2,11,12]. In fact, at high growth speeds, this ternary composite presents a finer microstructure than the one of binary alumina-based compounds, due to the presence of ZrO_2_, which makes more sluggish the liquid to three-phase ordered solid transition and reduces the growth length scale [13].

In spite of this, nowadays, the interest is partially shifting towards particulate composites. In fact, the unidirectional solidification from the melt poses some severe limitations in the size and shape of the components and is associated with high production costs. In addition, the eutectic composites are characterized by anisotropic physical and mechanical properties, which depend on the solidification direction [3,8]. All of these drawbacks can be easily overcome by the manufacturing of sintered polycrystalline materials. Once again, the binary Al_2_O_3_/YAG [14,15] and the ternary Al_2_O_3_/ZrO_2_/YAG [16,17] sintered composites seem promising for high-temperature structural applications [14,15,16].

This work aims at developing and characterizing a triphasic particulate composite, whose composition is Al_2_O_3_/5 vol% ZrO_2_/5 vol% YAG (AZY). While ceramic composite powders are commonly prepared by the traditional milling and mixing method [16], here, an innovative process has been exploited, already used by some of the authors in some previous works [18,19,20]. This method, named the surface coating route, implies the surface modification of alumina commercial powders by inorganic precursors of the second phases. Controlled thermal treatments allow the crystallization of the second-phase particles on the alumina powder surface.

The sintered composites are submitted to a mechanical characterization. In particular, for the first time, to our best knowledge, the high-temperature flexural behavior (from room temperature to 1500 °C) of this composite system is investigated. Thus, samples were submitted to increasing loads at different temperatures (from room temperature to 1500 °C), for assessing the temperature-induced modifications in the mechanical behavior. Further work could imply the use of different strain rates during the high-temperature tests, thus also allowing one to discuss the eventual creep behavior of the materials. Therefore, the results of this work, even if preliminary, will contribute to deepening the possible use of this material in high-temperature applications.

## 2. Results and Discussion

**Figure 1:**
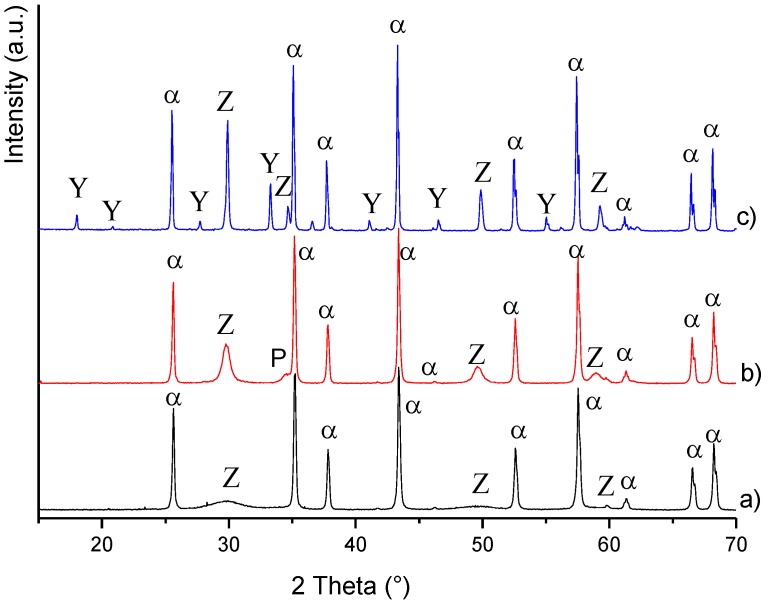
XRD patterns of Al_2_O_3_/5 vol% ZrO_2_/5 vol% YAG (AZY) calcined powders (α = α-Al_2_O_3_; Z = c-ZrO_2_; Y = YAG; P = perovskite YAlO_3_).

Figure 1 collects the X-ray diffraction (XRD) patterns of AZY powder calcined at 600 °C/1 h, 1050 °C/5 min and 1500 °C/3 h. At the lowest calcination temperature (pattern a), we can observe the crystallization of the cubic-ZrO_2_ phase (Zr_0.72_Y_0.28_O_1.862_, International Centre for Diffraction Data, ICDD No. 7-2112), besides the α-Al_2_O_3_ peaks (ICDD No. 46-1212). The powder calcined at 1050 °C (pattern b) showed a very similar diffraction pattern. Here, besides a slightly better crystallized cubic-ZrO_2_ phase, a small peak at 34.4 2θ can be detected, imputable to the crystallization of the perovskite YAlO_3_ phase (ICDD No. 70-1677). This secondary phase disappeared by increasing the calcination temperature: in fact, the YAG phase started to crystallize at 1300 °C [20], and after calcination at 1500 °C/3 h (pattern c), the powder was purely composed by the α-Al_2_O_3_, c-ZrO_2_ and YAG phases.

The powder calcined at 1050 °C/5 min was dispersed by ball-milling. After drying, green bodies were prepared by uniaxial pressing, as described in the Experimental Section. The samples were then pressureless sintered at 1500 °C/3 h, reaching full densification (average fired density of 99.7%).

**Figure 2:**
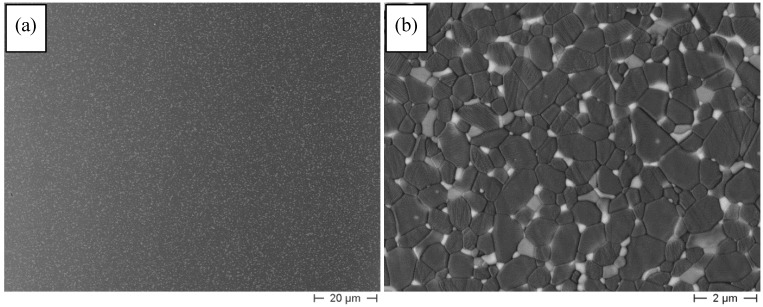
(**a**) Lower- and (**b**) higher-magnification Field Emission Scanning Electron Microscope (FESEM) images of AZY sintered at 1500 °C/3 h.

In Figure 2, we can observe the micrographs of the sintered AZY composite. By the lower-magnification image (Figure 2a), we can appreciate the fully dense and highly homogeneous microstructure. No observable flaws, nor residual pores can be detected in the sintered material. The three ceramic phases show a very good distribution in the composite structure. The higher magnification image (b) allows a deeper observation of the particle distribution and size. The phase contrast in the image allows recognizing the three crystalline phases: the darker phase can be attributed to α-Al_2_O_3_, the lighter one to ZrO_2_ and the gray one to YAG. Although both cubic-ZrO_2_ and YAG grains were predominantly located at the alumina grain boundaries, some ultra-fine particles were observed in intra-granular position (see the arrows in Figure 2b). Alumina grains are mainly equiaxial, and no abnormally grown grains were observed, due to the effective pinning exerted by the second-phase grains on the alumina grain boundaries.

Table 1 collects the average grain size of the three phases in the sintered composite. The results show an overall fine microstructure, made by a sub-micronic alumina matrix and ultra-fine YAG and ZrO_2_ grains.

**Table 1:** Average grain size of Al_2_O_3_, YAG and ZrO_2_ particles in the as-sintered material and in the sample submitted to the flexural test at 1500 °C.

Sample	Average Al_2_O_3_ Grain Size (µm)	Average YAG Grain Size (µm)	Average c-ZrO_2_ Grain Size (µm)
AZY as-sintered	0.82 ± 0.30	0.45 ± 0.15	0.28 ± 0.09
AZY after flexural test at 1500 °C	0.83 ± 0.31	0.45 ± 0.34	0.28 ± 0.09

**Figure 3:**
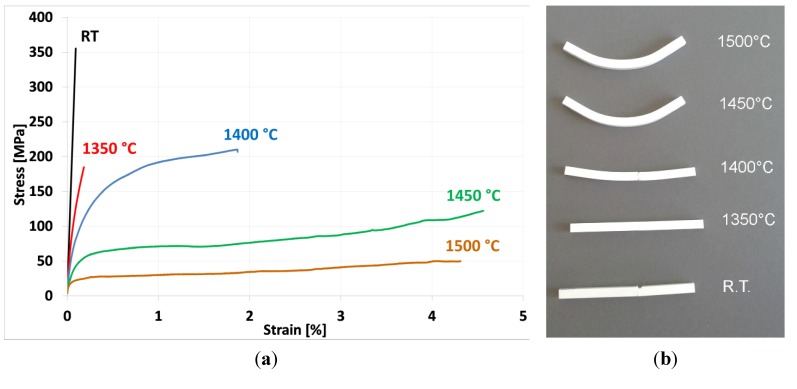
(**a**) Results of the flexural tests, carried out on the AZY samples at the different testing temperatures; (**b**) photograph of the samples, after the mechanical testing at increasing temperatures. RT: Room Temperature.

In Figure 3a, the stress-strain curves at the different testing temperatures are depicted.

The sample tested at room temperature shows a purely brittle behavior, with a flexural strength of about 320 MPa. As a comparison, Lack *et al.* [21] investigated the room-temperature mechanical behavior of alumina–YAG particulate composites, determining a flexural strength in the range 300–350 MPa, depending on the YAG content and sintering temperature. Slightly higher values (between about 350 and 450 MPa) were determined by Sommer *et al.* [22] for alumina–YAG composites, with a YAG content ranging between 5 and 20 vol%. Waku *et al.* [23] reported a flexural strength of about 420 MPa for an alumina–YAG composite having a eutectic composition. Concerning the triphasic AZY system, Oelgardt *et al.* [16] investigated a 45 vol% alumina–17 mol% ZrO_2_–38 mol% YAG composite and determined a flexural strength of about 280 MPa. Kim *et al.* [17] determined values in the range of 200–350 MPa for the 33 vol% alumina–33 vol% ZrO_2_–33 vol% YAG composite. Although limited, these previous data are in a good agreement with the current results.

By increasing the temperature of the bending test, we can observe a clear change in the material’s behavior. In fact, the samples tested at 1350 °C still showed an almost brittle behavior, whereas at 1400 °C, the samples showed a significant plastic deformation before failure. A limited temperature increase (50 °C) induced a remarkable change in the material’s mechanical behavior. At the highest testing temperatures (1450 °C and 1500 °C), the plastic deformation was so significant that it reached the maximum value allowed by the testing machine, but the samples were still unbroken.

Figure 3b shows a photograph of the samples tested at increasing temperatures, to underline their different mechanical behavior.

**Figure 4:**
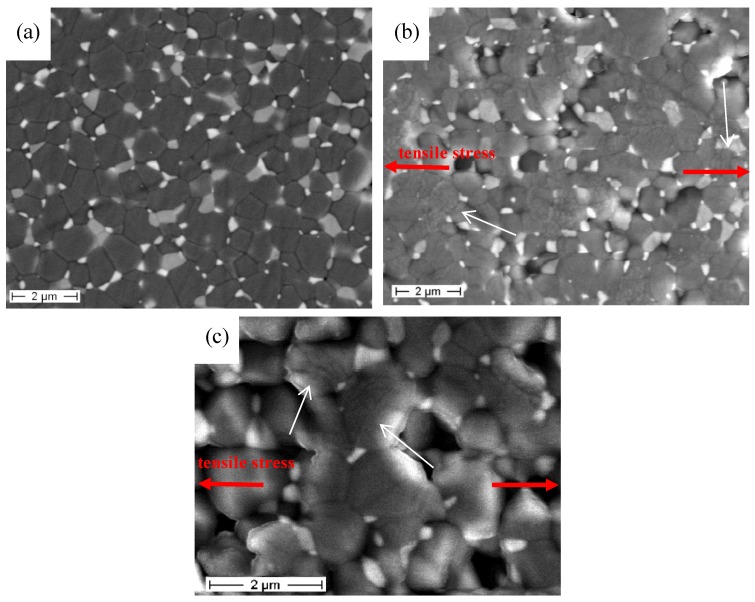
FESEM micrographs of AZY submitted to flexural testing at 1500 °C. (**a**) Un-loaded area; (**b**) lower- and (**c**) higher-magnification images of the area submitted to tensile stress (loading direction shown by red arrows; microcracks by white arrows).

As shown in Table 2, the flexural strength and the elastic modulus progressively decreased by increasing the testing temperature, whereas the elongation at break increased. The samples tested at 1450 °C and 1500 °C experienced the same deformation (of about 4.5%), but the former sample reached almost double the stress of the latter material. A direct comparison with previous literature data can be hardly made, since most of the previous works refer to different kinds of composites (melt-grown eutectics, instead of particulate composites), different testing apparatuses and conditions (creep tests, compressive tests, *etc*., instead of the high-temperature four-point bending test, with a short duration). In spite of this, a representative comparison can be made with the previous work of Waku *et al*. [23], showing the evolution of the flexural strength of alumina–YAG particulate composites as a function of temperature. In spite of the different instrumental set-up used in [23] (three-point flexural tests, on smaller samples), we can observe a comparable behavior, with a progressive decrease of the strength from the initial value (about 420 MPa, tested at room temperature) to about 50 MPa at 1600 °C. However, they observed a significant grain growth after testing at 1400 °C, contrary to our work, in which no appreciable microstructural change occurred during the high-temperature mechanical tests. In fact, as observed by FESEM, the main microstructural features (*i.e.*, alumina and second-phase grain size, second-phase distribution, grain morphology and orientation) were the same before and after the tests. A systematic observation allowed excluding the presence of elongated grains, deformed along the loading direction (red arrows in Figure 4). In fact, the microstructure was still composed by equiaxial grains, even after the high-temperature tests. This was imputed to the very short test duration (in the range of 20–200 s), not allowing the occurrence of possible microstructural modifications. For the sake of clarity, the average grain size of the alumina, ZrO_2_ and YAG phases, determined in the sample tested at 1500 °C, are collected in Table 1. We can see almost the same values of the as-sintered, untested specimen.

**Table 2:** Results of the flexural tests, carried out at the different testing temperatures.

Testing Temperature	Flexural Strength (MPa)	Elastic Modulus (GPa)	Elongation at Break (%)
25 °C	320.9 ± 48.6	365.6 ± 12.9	0.09 ± 0.01
1350 °C	174.0 ± 15.1	219.4 ± 13.4	0.15 ± 0.04
1400 °C	202.4 ± 11.2	136.7 ± 17.0	1.64 ± 0.3
1450 °C	120.7 ± 1.8	98.1 ± 19.5	n.d. *
1500 °C	52.8 ± 4.7	93.8 ± 0.8	n.d. *
1450 °C (sample heat treated 1 h at 1700 °C)	105.3 ± 5.6	125.4 ± 18.9	0.10 ± 0.03

* Not determined, since these samples reached the maximum deformation allowed in the testing machine, without failure.

However, in the 1450 °C- and 1500 °C-tested materials, some difference arose by comparing the un-loaded areas (outside the supports) with those submitted to the highest tensile stress (near the surface, between the loading pins, deformed over 4%), as shown in Figure 4, where some microcracks (mainly in intra-granular positions; see the white arrows in Figure 4b,c) were observed. In addition, in the polished, tensile-stressed region, a severe grain pull-out phenomenon was observed, which was completely absent in the un-loaded areas.

According to these observations, the change of the mechanical behavior within the temperature could be imputed to some thermally-induced deformation mechanisms, such as grain boundary sliding, and not to a microstructural change induced by the prolonged heating of the samples. Such a deformation mechanism seems connected with the generation of microcracks, which could be responsible for the grain pull-out observed on the mostly stressed surfaces. However, in a future work, a deeper observation by Transmission Electron Microscopy (TEM) will be carried out, in order to better highlight such microstructural features and establish meaningful microstructure-property correlations during the high-temperature tests. Furthermore, this technique will allow recognizing, if present, deformation structures in the plastically deformed grains, as already observed in alumina-YAG eutectic composites [24].

In order to deepen the thermal stability of the samples and the relationship between microstructure and mechanical properties, pellets sintered at 1500 °C/3 h were re-heated in the 1550–1700 °C range and submitted to a microstructural and mechanical characterization, by dynamic indentation.

**Figure 5:**
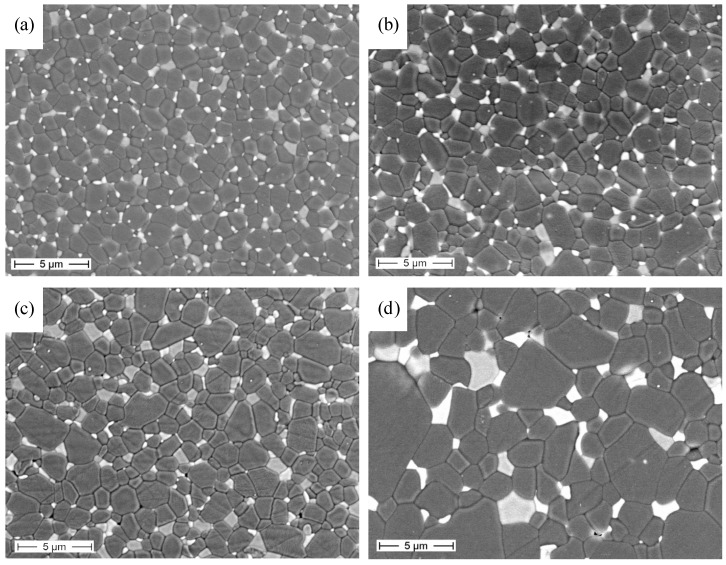
FESEM micrographs of AZY sintered at 1500 °C/3 h and re-heated at (**a**) 1550 °C; (**b**) 1600 °C; (**c**) 1650 °C and (**d**) 1700 °C for 1 h.

In Figure 5, the microstructures of the fired samples are depicted.

**Figure 6:**
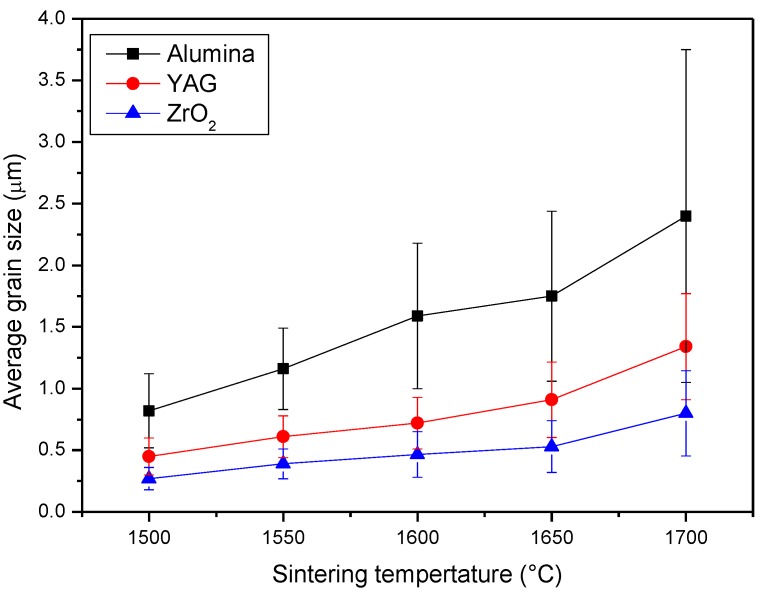
Grain size evolution as a function of the firing temperature.

Up to 1650 °C, we can see that the materials underwent a limited grain growth; the microstructure was still very homogeneous, made by equiaxed alumina and fine second-phase grains, showing that the pinning effect exerted by the second phases was still effective up to this temperature. On the opposite side, at 1700 °C, a significant grain growth occurred, involving both the alumina and the second phases. The morphology of the alumina grains partially changed, since several elongated particles were observed besides equiaxed ones. An increased fraction of intragranular second-phase grains was observed, as well. The grain size evolution is better evidenced in Figure 6: at 1700 °C, both the average value of the alumina size and the related standard deviation increase, thus attesting to a less homogeneous size distribution of the matrix grains.

**Figure 7:**
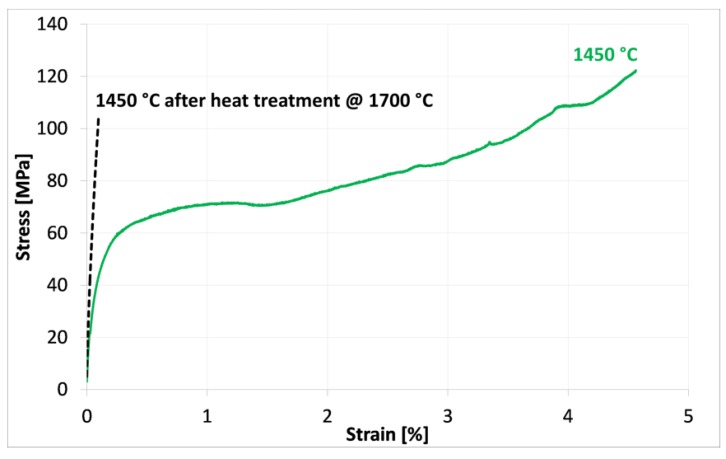
Results of the flexural tests at 1450 °C, carried out on the as-sintered sample (green curve) and on the same material previously treated at 1700 °C (black curve).

According to this result, some samples were treated at 1700 °C for 1 h (in order to promote grain growth) and then submitted again to the flexural tests at 1450 °C. As shown in Figure 7, the increase in grain size for these samples causes a strong modification of the mechanical behavior, with an evident reduction of the plastic deformation. This result strengthens the role of the grain boundary sliding phenomena, promoted in the ultra-fine-grained microstructure, but very limited in the coarsened material.

Dynamic indentations were carried out at room temperature, both on the mechanically-tested samples (measures performed in unstressed zones of the specimens) and on the heat-treated ones up to 1700 °C. The Young’s modulus and microhardness were evaluated.

**Figure 8:**
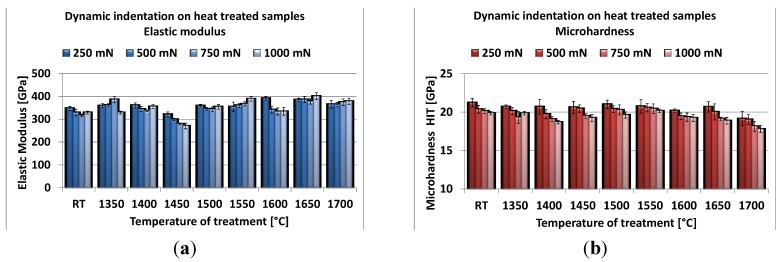
Young’s modulus (**a**) and microhardness (**b**) of AZY samples, submitted to flexural tests at increasing temperatures.

The elastic modulus (Figure 8a) was almost constant, without a clear dependence on the exposure temperature (RT–1500 °C) and the indentation load. The average values are close to the Young’s modulus determined for the room-temperature tested material (about 370 GPa, as reported in Table 2); also, the samples heat treated at higher temperatures (1550–1700 °C) do not show a significant variation in the measured elastic moduli. The analysis of the microstructure and grain size evolution could help to explain the lack of Young’s modulus variation. Up to 1650 °C, the samples underwent a limited grain size modification; at 1700 °C, when a significant grain growth occurred, the grain size still remains lower than the indentation area (theoretically about 12.5 µm^2^ with a load of 0.25 N and a hardness of 20 GPa) and, above all, much smaller than the elastoplastic indentation volume [25]. For this reason, the indentations involve, also in the specimens exposed to 1700 °C, a multiphasic portion of material, and the calculated elastic properties take into account the contribution of all of the different phases present in the samples.

Figure 8b shows the evolution of the microhardness as a function of the applied load and testing temperature. The indentation hardness (HIT) is defined as the ratio between the maximum applied load and the projected contact area. We can observe that for each temperature, the hardness decreases by increasing the applied load, showing an indentation size effect in these materials. On the opposite side, the testing temperature had a minor effect on the samples’ hardness up to 1650 °C, since almost the same value was obtained for all samples: only the sample re-heated at 1700 °C exhibits an appreciable hardness decrease, probably due to the significant grain growth occurring at this temperature.

These data strengthen the observation that no major microstructural modifications occurred in the materials during the high-temperature exposure in the range of RT–1650 °C.

## 3. Experimental Section

Al_2_O_3_/5 vol% ZrO_2_/5 vol% YAG composite powder (AZY) was prepared starting from a commercial α-Al_2_O_3_ powder (TM-DAR TAIMICRON, supplied by Taimei Chemical Co., Tokyo, Japan). This powder is highly pure (>99.99%), characterized by a specific surface area of 14.5 m^2^/g and by an average particle size of 150 nm.

An aqueous suspension of the alumina powder was dispersed by ball-milling for 15 h. Then, it was mixed with a salts solution, containing YCl_3_·6H_2_O (Sigma-Aldrich, purity: 99.99%, St. Louis, MO, USA) and ZrCl_4_ (Fluka, purity > 98%, St. Gallen, Switzerland). Tribasic ammonium citrate (Sigma-Aldrich, >97% purity) was also added, in order to increase the pH of the zirconium chloride solution from 1 to about 4.5. The amount of the yttrium chloride was calculated in order to both develop the YAG phase (through a solid state reaction with alumina [26]) and to fully stabilize zirconia as the cubic phase. The modified suspension was then spray dried (Buchi, Mini spray dryer B-290, Flawil, Switzerland). More details about the elaboration process can be found elsewhere [18,19,20]. According to some previous results [20], the powder was calcined at 600 °C, for 1 h, with the aim of inducing the decomposition of the synthesis by-products and the crystallization of the ZrO_2_ grains on the surface of the alumina particles. The powder was then submitted to a second thermal cycle, at 1050 °C for 5 min [18], in order to induce the crystallization of the yttrium aluminate phase. The phase evolution was investigated by X-ray diffraction (XRD, Philips PW 1710, Eindhoren, The Netherlands).

The calcined powder was dispersed by ball-milling for about 20 h. After drying, the powder was pressed in pellets or in bars, suitable for the mechanical test, and sintered at 1500 °C, for 3 h, at 2 °C/min.

In order to investigate the microstructural evolution as a function of the sintering temperature, some sintered samples were re-heated at higher temperatures (in the range of 1550–1700 °C, for 1 h).

The fired density was evaluated by the Archimedes’ method and referred to the theoretical density of the composite (4.11 g/cm^3^), calculated by the rule of mixture. For the calculation, theoretical densities of 3.99, 5.82 and 4.55 g/cm^3^ were used for α-alumina, c-ZrO_2_ and YAG, respectively.

The microstructure of the sintered samples (polished and thermally-etched surfaces) was observed by FESEM (Hitachi S4000, High-Technologies Co., Tokyo, Japan). The grain size of the alumina matrix and of the two second phases was determined by image analysis (Scandium Soft imaging system software, Olympus, Münster, Germany) carried out on several micrographs.

Bars (final dimensions of 45 × 4 × 1.8 mm^3^) were polished and tested by four-point bending tests at various temperatures, *i.e.*, room temperature, 1350 °C, 1400 °C, 1450 °C and 1500 °C, according to ASTM C1161 (room temperature) and C1211 (high temperature) [27,28]. The samples were heated at 15 °C/min up to the final temperature, maintained at this temperature for 1 h and then submitted to the flexural test. For each temperature, three samples were tested. The SiC testing assembly was placed inside a furnace (Maytec GmbH, Singen, Germany) able to reach 1500 °C, which was connected to a Zwick-Roell Z2.5 universal testing machine (Zwick GmbH, Ulm, Germany) in order to measure the force applied to the sample. The test was conducted using a constant crosshead speed of 0.5 mm/min. This value is suggested by the ASTM standards in order to minimize the test time and creep influence. The test duration varied from about 20 to 200 s (depending on the test temperature and maximum deformation reached during the test), according to the standard recommendations. A properly designed displacement transducer, pushing against the tension face of the sample using three alumina rods, is able to reconstruct and to measure the sample curvature and, afterwards, the deformation [29,30,31]. Finally, the stress-deformation curves, the elastic constants and the mechanical properties were assessed. Young’s modulus calculation was carried out using a regression of the stress-strain curve between 10 and 25 MPa.

The evaluation of the microhardness and Young’s modulus by dynamic indentation (ISO 14577:2007) [32] was performed, using an instrumented indentation system (Nanotest, MicroMaterials Ltd., Wrexham, UK). Maximum loads of 250, 500, 750 and 1000 mN were applied for 10 s, using a Berkovich 3-sided indenter. Both loading and unloading velocity was fixed at 100 mN/s. Post-test analysis of the load/penetration curves was carried out using the approach described by Oliver and Pharr [33,34]; the reduced elastic modulus was calculated considering the unloading curve and applying the following equation:

$$\frac{1}{E_{r}}=\frac{\left(1-{n_{s}}^{2}\right)}{E_{s}}+\frac{\left(1-{n_{i}}^{2}\right)}{E_{i}}$$

where *n_s_* is the sample’s Poisson ratio, *n_i_* and *E_i_* are the diamond indenter’s Poisson ratio and elastic modulus (0.07 and 1141 GPa, respectively).

Indentation tests were carried out at room temperature, both on as-sintered and heat-treated samples.

## 4. Conclusions

Al_2_O_3_/5 vol% ZrO_2_/5 vol% YAG composite powders have been prepared by an innovative surface coating route, consisting of the surface modification of a commercial α-Al_2_O_3_ powder with the inorganic precursors of the second phases. The composite powders, after die-pressing and pressureless sintering at 1500 °C for 3 h, gave rise to fully dense sintered materials.

The elaboration process employed here was successful at producing composites with a highly homogenous microstructure, characterized by an optimal distribution of ultra-fine ZrO_2_ and YAG grains inside a sub-micronic alumina matrix.

The mechanical behavior of the material showed a strong dependence on the testing temperature: the samples submitted to four-point flexural test at room temperature and at 1350 °C showed a brittle behavior, whereas at 1400 °C, the materials experienced a significant inelastic deformation before failure. The samples tested at 1450 °C and 1500 °C reached the maximum deformation level allowed by the testing machine (about 4.5%) without breaking. At the same time, both the flexural strength and the elastic modulus decreased by increasing the testing temperature. Microstructural observations confirmed the stability of the samples after the high-temperature tests.

Sintered samples, re-heated in the 1550–1700 °C range, showed a limited grain growth up to 1650 °C, with still a very homogeneous and fine microstructure. On the opposite side, at 1700 °C, a significant grain growth occurred, which involved both the alumina and the second phases. The Young’s modulus and micro-hardness values provided by the instrumented indentation tests did not show a significant dependence on the heat treatment temperatures: only the sample treated at 1700 °C exhibits an appreciable reduction in hardness, probably induced by the grain growth occurring at this temperature. Bending tests conducted on samples heat treated at 1700 °C show a strong influence of thermal treatment on the mechanical behavior, with a significant reduction of inelastic deformation at high temperatures. This was thus reasonably imputed to a limitation of the grain boundary sliding phenomena in the coarse-grained microstructure, as compared to the ultra-fine one.

In light of the reported results, some interesting perspectives can be highlighted: the ceramic composite showed an effective mechanism of grain growth suppression induced by the homogeneous dispersion of ultra-fine YAG and ZrO_2_ grains in the alumina matrix; this behavior could make the investigated material suitable for high temperature applications in which the excellent stability of the mechanical properties is required. The benefit of using a fine-grained material, also for exposure to high-temperatures, is here assessed. In fact, the plastic behavior exhibited at temperatures higher than 1400 °C could indicate the possibility of manufacturing complex-shaped components by the high temperature plastic forming processes, still maintaining the initial microstructure. Further investigations have to be performed in order to better understand the properties of Al_2_O_3_–ZrO_2_–Y_3_Al_5_O_12_ composites: particularly, the microstructure modifications induced by long-time heat treatments and the possible high temperature superplastic behavior have to be evaluated, focusing also on the creep resistance of this material.
